# Comparison of Surgical Outcomes of Hysterectomy by Vaginal Natural Orifice Transluminal Endoscopic Surgery (vNOTES) versus Single-Port Access (SPA) Surgery

**DOI:** 10.3390/jpm12060875

**Published:** 2022-05-26

**Authors:** Joseph J. Noh, Myeong-Seon Kim, Jun-Hyeok Kang, Ji-Hee Jung, Chi-Son Chang, Jungeun Jeon, Tae-Joong Kim

**Affiliations:** 1 Gynecologic Cancer Center, Department of Obstetrics and Gynecology, Samsung Medical Center, Sungkyunkwan University School of Medicine, Seoul 06351, Korea; josephnoh.medicine@gmail.com (J.J.N.); jihee0627.jung@samsung.com (J.-H.J.); cs.chang@samsung.com (C.-S.C.); j0716.jeon@samsung.com (J.J.); 2 Division of Gynecologic Oncology, Department of Obstetrics and Gynecology, St. Vincent’s Hospital, College of Medicine, The Catholic University of Korea, Seoul 03083, Korea; mseon.kim@outlook.com; 3 Uijeongbu Eulji Medical Center, Department of Obstetrics and Gynecology, Eulji University School of Medicine, Uijeongbu 11759, Korea; junhyuck1985@gmail.com

**Keywords:** natural orifice transluminal endoscopic surgery, single-port access surgery, single-incision laparoscopic surgery, minimally invasive surgery, gynecology, hysterectomy

## Abstract

Single-port access (SPA) laparoscopic surgery and vaginal natural orifice transluminal endoscopic surgery (vNOTES) have many advantages. The objective of the present study is to compare patient characteristics, operative details, and postoperative outcomes between the two surgical methods. Patients who were planned to undergo vNOTES or SPA laparoscopic surgery between April 2020 and June 2021 were prospectively enrolled. The surgical method was determined by a single surgeon after imaging results evaluation and a physical exam. Those who had favorable pelvic conditions without any evidence of adhesion were scheduled for vNOTES. A total of 33 patients underwent a vNOTES hysterectomy while 40 patients received a SPA laparoscopic hysterectomy. All surgeries were performed by one surgeon. The proportion of the patients who had a history of vaginal delivery was significantly higher in the vNOTES group. The operative time for port installation was significantly longer in the vNOTES group, but the total operative time was shorter compared to the SPA group. The postoperative pain scores 12 h after the operations were also significantly lower in the vNOTES group. Other surgical outcomes were comparable between the two groups. The present study demonstrated that the early operative outcomes of vNOTES hysterectomy were comparable to those of SPA hysterectomy. It also highlights the importance of adequate patient selection when determining surgical methods based on imaging results and physical examinations.

## 1. Introduction

Minimally invasive surgery (MIS) has gained popularity in the field of gynecologic surgery since the late 1980s and early 1990s. Among the various MIS surgical approaches, single-port access (SPA) laparoscopic surgery and natural orifice transluminal endoscopic surgery (NOTES) have gained attention for their many advantages. The benefits of SPA laparoscopic surgery have been observed in postoperative pain scores, duration of hospital stay, and cosmetic satisfaction as compared to standard laparoscopic surgery [1]. It has also demonstrated clinical outcomes comparable to those of standard laparoscopic surgery and overall low rates of major perioperative morbidity [2,3,4]. NOTES, which utilizes the natural orifices such as the mouth, vagina, urethra, and rectum as an accessible entry into the peritoneal cavity, was first described in 2004 [5] and the feasibility and safety of vaginal NOTES (vNOTES) were first demonstrated in 2012 [6]. Since then, this surgical approach has been increasingly adopted as a minimally invasive modality for various gynecological surgeries, including hysterectomy, myomectomy, adnexectomy, omentectomy, and uterosacral ligament suspension [7,8,9,10]. The advantages of vNOTES include reduced postoperative pain, faster postoperative recovery, decreased postoperative wound infections, and outstanding cosmetic results [11].

Data regarding vNOTES are still insufficient, which may limit the availability of training and the greater diversity of operative techniques by different surgeons. Furthermore, a comparative investigation of the surgical outcomes of vNOTES and SPA laparoscopic surgery has not been conducted to date. To the best of our knowledge, only two studies have compared the operative outcomes of vNOTES and SPA laparoscopic surgery [12,13]. The present study aimed to compare the two surgical methods. The primary outcomes of the present study are postoperative complication rates. We also examined postoperative pain and length of surgical time as secondary outcomes.

## 2. Materials and Methods

### 2.1. Patients

The present study included patients who were diagnosed with benign gynecological diseases and scheduled for surgery between April 2020 and June 2021. The inclusion criteria of the study were patients scheduled to undergo vNOTES or SPA laparoscopic surgery. The exclusion criteria were those with any preoperative assessment results that were suggestive of malignancy and patients who were not candidates for minimally invasive surgery (e.g., patients with a uterus greater than 15 cm in size). Before determining the surgical approach, a detailed collection of previous medical/operative history, related symptoms, and imaging results, including ultrasonography, computed tomography (CT), or magnetic resonance imaging (MRI), were analyzed. The surgeon (TJ Kim) performed a bimanual pelvic examination to determine the width of the vaginal canal and the size and movability of the uterus. Patients who demonstrated any evidence of pelvic adhesion during this procedure or who had a narrow vaginal canal underwent SPA laparoscopic surgery, whereas those with favorable pelvic conditions underwent vNOTES.

### 2.2. Operative Techniques

All the patients underwent the same standard preparation before surgery. Prophylactic antibiotics were administered 30 min before incision. After general anesthesia and endotracheal intubation, the patients were placed in the Trendelenburg position with lithotomy. A 12-Fr Foley catheter was then inserted. For patients who underwent vNOTES, surgical procedures were performed as described previously [14]. In brief, after incising circumferentially at the junction of the vagina and cervix as is generally performed in vaginal hysterectomy, the vesicovaginal space is incised by blunt and sharp dissection while culdotomy is performed similarly. Both the uterosacral ligaments and uterine arteries were ligated using absorbable suture materials. Subsequently, a commercial single-port platform was installed (LapSingle, Sejong Medical, Inc., Paju, South Korea). CO_2_ gas at 10 or 11 mmHg was used to establish pneumoperitoneum, which is lower than that used in conventional laparoscopic surgery. We used a 30°-angled 5 mm endoscopy (Karl Storz SE & Co. KG, Tuttlingen, Germany) and laparoscopic instruments, such as suction and irrigator, short grasper (ENDOPATH^TM^ Grasper 5 mm, Ethicon, Inc., Somerville, NJ, USA), and Enseal^TM^ G2 Tissue Sealer (45 cm length and curved tip, Ethicon, Inc., Somerville, NJ, USA). The procedures described in our previous study were adopted for SPA laparoscopic surgery [15]. After incising the skin at approximately 2.0–2.5 cm, subcutaneous tissue and anterior abdominal fascia were opened by Bovie electrocauterization in 40 W monopolar coagulation mode (Medtronic, Inc., Minneapolis, MN, USA) using the open Hasson technique. A commercial single-port platform was installed, including One Port^TM^ (LapaKorea, Inc., Seoul, Korea) or LapSingle^TM^ (Sejong Medical, Inc., Paju, Korea). The CO_2_ pneumoperitoneum was maintained at 13 mmHg throughout the surgery. The instruments used during the operations included monopolar scissors, laparoscopic energy devices such as Enseal^TM^ G2 Tissue Sealer (Ethicon, Inc., Somerville, NJ, USA), myoma screws, laparoscopic needle holders, and articulating graspers (Roticulator^TM^, Covidien, Inc., Norwalk, CT, USA).

### 2.3. Data

The baseline characteristics of the patients, including the results of the preoperative imaging studies, were collected. The primary outcome of the present study was postoperative complication rates. Intraoperative complications such as bowel, ureter, and bladder injury and immediate postoperative complications (occurring within 90 days of surgery), including ileus, wound dehiscence, surgical site infection, and transfusion, were monitored. One of the secondary outcomes of the present study was postoperative pain severity. Postoperative pain was measured by asking the patients to rate their pain using the visual analog scale (VAS) that was posted at each patient’s bedside. The surgeon specifically asked the patients to provide a number between 0 and 10 indicating how severe their pain was each time. Surgical time was also measured as a secondary outcome of the study. The total operative time was defined as the time from the beginning of the skin (or vaginal) incision to the completion of skin (or vaginal) closure. The time required to complete each step, such as the time for port installation, ligation of the uterine arteries, and suturing of the vagina, was measured separately. Other measures included estimated blood loss (EBL), hemoglobin changes between preoperative and postoperative status, number and types of analgesics requested by the patients postoperatively, and length of postoperative hospital stay. EBL was calculated by subtracting the volume of irrigating fluid from the volume of total fluid collected in the suction apparatus after surgery. A single pathologist measured the total weight of the resected uterus. All patients received patient-controlled analgesia (PCA), which was composed of fentanyl 1500 mcg with nefopam HCL 80 mg, for 24 h postoperatively along with oral zaltoprofen 80 mg three times a day until discharge. Additional analgesic medications were available at the patients’ request. Preoperative hemoglobin levels were measured within 1 month before the operation, and postoperative hemoglobin levels were measured on postoperative day (POD) 1.

### 2.4. Statistical Analyses

The data are expressed as mean ± standard deviation for continuous variables. Statistical significance was determined using Fisher’s exact test for dichotomous variables and independent Student’s *t*-test for continuous variables. Statistical significance was set at *p* < 0.05. Statistical calculations were performed using R version 3.6.2. (Vienna, Austria; http://www.R-project.org/, accessed on 1 March 2022).

### 2.5. Ethics Approval

All procedures were performed in accordance with the ethical standards of the institution and the 1964 Helsinki Declaration (and its later amendments). Approvals were obtained from the Samsung Medical Center institutional review board.

## 3. Results

**Figure 1:**
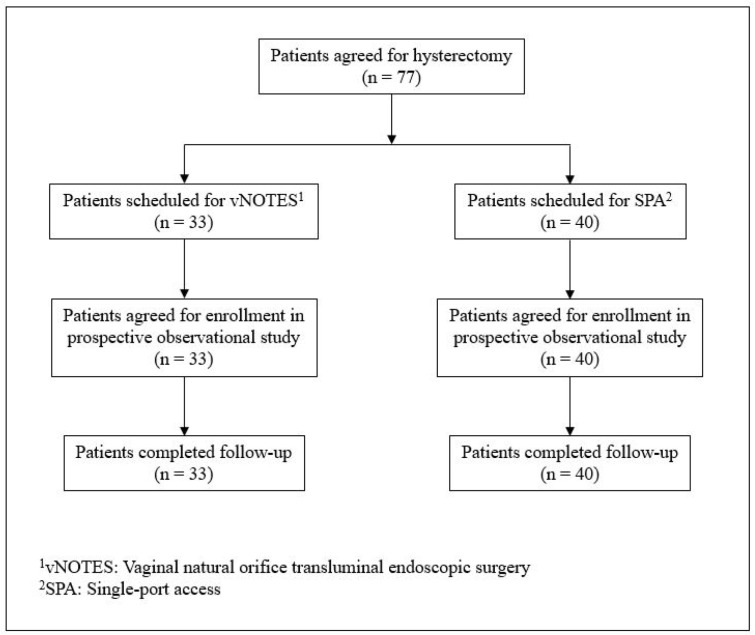
Study flowchart.

A total of 33 patients underwent vNOTES hysterectomy, while 40 underwent SPA laparoscopic hysterectomy (Figure 1). All surgeries were performed by one surgeon (TJ Kim), who had performed over 50 vNOTES and 2500 SPA laparoscopic surgeries. The baseline characteristics of the patients in the two groups are described in Table 1. The proportion of the patients who had a history of vaginal delivery was significantly higher in the vNOTES group (93.5% vs. 45.7%, *p*-value < 0.001). Otherwise, the two groups were comparable with respect to age, parity, body mass index (BMI), history of previous abdominal surgery, and preoperative ultrasonography findings. The size and position of the uterus on preoperative ultrasonography did not differ between the two groups. The surgical information and postoperative outcomes are described in Table 2. EBL, hemoglobin changes, duration of hospital stay, and weight of the extracted specimen did not show any statistical difference between the two groups. Patients who underwent vNOTES demonstrated significantly less pain 12 h after surgery compared to those who underwent SPA laparoscopic surgery. The difference in postoperative pain between the two groups disappeared 24 h after surgery. The proportion of patients who requested rescue analgesics in addition to their PCA was evaluated; however, this did not differ between the two groups. No intraoperative complications or instances of conversion from MIS to laparotomy were observed in either group. However, one patient who underwent vNOTES hysterectomy and bilateral salpingectomy for leiomyoma (7 cm, uterine weight 205 g) was found to have a ureteral injury, which required ureteroneocystostomy with a psoas hitch and double-J catheterization. The patient was discharged on POD 2 after vNOTES surgery uneventfully; however, she came to the outpatient clinic on POD 8 with abdominal distension and profuse urinoma.

Time measurements for each surgical procedure were compared between the two groups. As can be seen in the table (Table 3), completion of port installation took significantly longer in the vNOTES group (18 min vs. 5 min, median, *p*-value < 0.001). However, once the installation of the port was completed it took less time to perform the rest of the procedures in the vNOTES group than in the SPA group. The time for vaginal closure was also shorter in the vNOTES group (6 min vs. 12.5 min, median, *p*-value < 0.001). The median total operative time was 64 min for the vNOTES group and 82 min for the SPA group, showing a statistically significant difference (*p*-value < 0.001).

## 4. Discussion

The present study demonstrated that the early operative outcomes of vNOTES hysterectomy are comparable to those of SPA hysterectomy. It was also found that patients who underwent vNOTES experienced less postoperative pain than those who underwent SPA laparoscopic surgery, and the total operative time of vNOTES was shorter than that of SPA laparoscopic surgery. To the best of our knowledge, the present study is the third to compare the surgical outcomes of vNOTES and SPA hysterectomy [12,13]. Both previous studies have demonstrated comparable surgical outcomes of vNOTES to those of SPA laparoscopic surgery, but those studies were conducted retrospectively whereas the present study was designed as a prospective cohort study. Furthermore, the present study analyzed operative time by each surgical procedure, thereby providing detailed comparisons between the two surgical methods.

Previous studies have reported that the advantages of vNOTES include reduced postoperative pain, faster recovery, decreased postoperative wound infection rates, and outstanding cosmetic results [16]. It has also been reported that manual suturing of the vaginal vault in vNOTES could be combined with counter prolapse interventions to reduce the need for future surgical interventions [17]. The results of the present study are consistent with those of previous studies. Although the frequency of rescue analgesics requested by the patients did not differ between the vNOTES and SPA laparoscopic surgery patients, the initial postoperative pain levels measured by NRS were significantly lower in the vNOTES patients. The total operative time was also shorter in patients who underwent vNOTES than in those who underwent SPA laparoscopic surgery. The authors previously analyzed the learning curve for port installation in vNOTES [14]. It was demonstrated that the learning curve of port installation and that of total operation dropped sharply after the first five cases and then reached close to the proficiency level by the 10th case. Although it took longer to complete port installation in vNOTES than in SPA laparoscopic surgery in the present study, the total operation time was significantly shorter in the vNOTES group. The time taken for each surgical step, such as ligation of the uterine arteries, colpotomy, and vaginal closure, was shorter in the patients who underwent vNOTES. In addition to not having to incise the umbilicus, which has more nerve endings and sensory innervations than the vaginal fornix [18], this shorter duration of surgery may have contributed to the lower postoperative pain felt by the vNOTES patients because of the shorter exposure duration of CO_2_ during vNOTES. Prolonged exposure to CO_2_ during laparoscopic surgery is known to cause shoulder and upper back pain caused by diaphragmatic irritation by gas [19].

**Figure 2:**
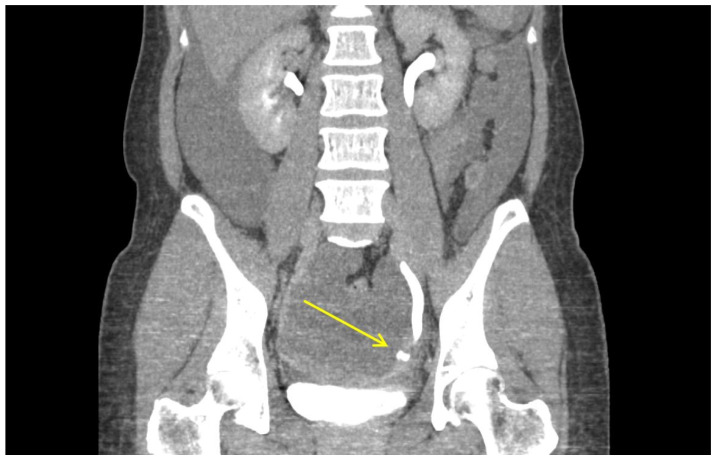
Sagittal image of urography CT (computed tomography) showing left distal ureteral contrast leakage with profuse amount of fluid collection in the abdominal cavity.

**Figure 3:**
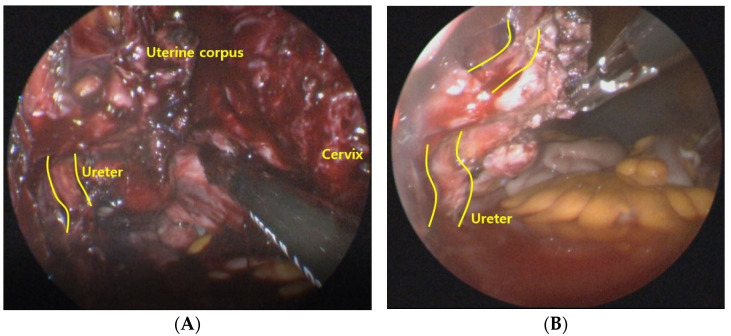
Image of the right ureter during vNOTES hysterectomy (**A**) when the uterus was still attached and (**B**) when the uterus was completely removed.

In terms of postoperative complications, neither the vNOTES nor the SPA group had vaginal vault complications. The vaginal closures of all patients included in this study were performed using a 23 cm, 2-0 knotless, delayed absorbable barbed suture material (Monofix^TM^, Samyang Biopharmaceuticals, Inc., Seoul, South Korea). This suture material is composed of a monofilament, which is less prone to colonization and discharge. None of the patients in either group complained of postoperative vaginal bleeding, discharge, dehiscence, or surgical site infection. However, as described above, there was one patient in whom the distal ureter was injured during the operation (Table 4 and Figure 2). Although previous studies have proposed better visualization of pelvic structures as one of the benefits of vNOTES [20,21], vNOTES carries the risk of injuring the urinary tract during surgery. A relatively quick envelopment of the surgical fume in the surgeon’s view due to the small pelvic space and blood oozing from the paracervical tissue due to continued blood supply from the gonadal vessels to the uterus, even after ligation of the uterine arteries, makes it susceptible to ureteral injury. In addition to these inherent characteristics of vNOTES surgery, the ureteral injury case described above was thought to be due to the surgeon’s approach, which was too lateral when ligating the uterine artery and the cardinal ligament. Blunt dissection and colpotomy, which rely solely on the surgeon’s palpation and tactile sense, also increase the risk of bladder and ureteral injuries. The best way to prevent such injuries is clear visualization of the important anatomical structures. It is feasible to visualize the ureter during vNOTES and avoid inadvertent damage even when the uterus has not been completely detached (Figure 3). Opening the anterior peritoneal leaf of the remaining cardinal ligament permits visualization.

The benefits of vNOTES may be maximized in specific patient populations. The wider shape of the pelvis in Western populations compared to Asian populations may allow for an easier surgical approach by surgeons through the vagina. The benefits of reduced port site herniation rates by approaching through the vagina instead of the umbilicus may also be expected in Western populations, who generally have a higher BMI compared to Asian populations. A high BMI is an important risk factor for postoperative port-site herniation.

vNOTES and SPA laparoscopic surgery both come with benefits and risks. In the present study, a single experienced surgeon thoroughly examined each patient preoperatively to determine the best surgical approach. Patients with a narrower pelvic space or who were expected to have adhesion in the intra-abdominal cavity were more likely to undergo SPA laparoscopy than those who underwent vNOTES. The criteria for adequate candidates for vNOTES were stricter than those for SPA laparoscopic surgery. Choosing between the two surgical methods based on preoperative examinations is a critical step and requires abundant experience from surgeons. Simultaneously, the fact that the surgeon of the present study decided which surgical approach to take for each patient may limit the generalizability of the results of this study. Patients who received vNOTES had more movable and non-adhered pelvic organs; therefore, they were generally easier to treat surgically compared to those who underwent SPA laparoscopic surgery. There is a need for further prospective data collection to continuously evaluate the safety and effectiveness of vNOTES. Patients’ perceptions, apprehensions, and prejudices regarding these surgical techniques that are still prevalent in many regions must be evaluated [22,23].

## Figures and Tables

**Table 1:** Baseline characteristics of the patients in the vNOTES and SPA groups.

	vNOTES (*n* = 33)	SPA (*n* = 40)	*p*-Value
**Age** (years)	48.0 ± 4.1	47.5 ± 4.7	0.615
**Parity ** 0 1 2 3	2 (6.1%) 7 (21.2%) 22 (66.7%) 2 (6.1%)	5 (12.5%) 11 (27.5%) 23 (57.5%) 1 (2.5%)	0.598
**Menopause ** No Yes	30 (90.9%) 3 (9.1%)	38 (95.0%) 2 (5.0%)	0.823
**BMI**^a^ (kg/m^2^)	22.4 (21.1, 24.2)	23.8 (21.3, 26.0)	0.091
**History of vaginal delivery ** No Yes	2 (6.5%) 29 (93.5%)	19 (54.3%) 16 (45.7%)	< 0.001
**History of previous abdominal surgery ** Appendectomy, laparotomy Appendectomy, laparoscopy Cholecystectomy, laparotomy Cholecystectomy, laparoscopy Myomectomy, laparoscopy Myomectomy, laparotomy Salpingectomy, laparoscopy HIFU ^a^	1 (3.0%) 3 (9.1%) 0 0 2 (6.1%) 0 1 (3.0%) 0	0 2 (5.0%) 1 (2.5%) 1 (2.5%) 1 (2.5%) 1 (2.5%) 2 (5.0%) 1 (2.5%)	0.480
**Size of the uterus** (cm) Length Anterior-posterior (AP)	10.2 ± 2.1 6.9 ± 1.9	10.9 ± 2.5 8.0 ± 2.7	0.245 0.084

^a^ BMI: body mass index, HIFU: high-intensity focused ultrasound.

**Table 2:** Surgical and post-operative outcomes of the vNOTES and SPA groups.

	vNOTES (*n* = 33)	SPA (*n* = 40)	*p*-Value
**Operation**^a^ SPA-TLH, US SPA-TLH, BS SPA-TLH, USO, US SPA-TLH, BSO vNOTES-H, US vNOTES-H, BS vNOTES-H, USO, US vNOTES-H, BSO	0 0 0 0 1 (3.0%) 25 (75.8%) 2 (6.0%) 5 (15.2%)	2 (5.0%) 23 (27.5%) 7 (17.5%) 8 (20.0%) 0 0 0 0	N/A
**Estimated blood loss** (ml)	150 (50, 200)	100 (90, 200)	0.446
**Hemoglobin changes** (g/dL) ^b^	−1.3 (−2.1, −0.8)	−1.7 (−2.6, −1.0)	0.136
**Post-operative hospital stay** (days) 1 2 3	1 (3.0%) 29 (87.9%) 3 (9.1%)	0 39 (97.5%) 1 (2.5%)	0.243
**Post-operative diagnosis ** Uterine leiomyoma Adenomyosis Others	23 (69.7%) 5 (15.2%) 5 (15.2%)	26 (65.0%) 11 (27.5%) 3 (7.5%)	0.250
**Median uterine weight** (g)	317.9 ± 161.1	408.8 ± 252.3	0.095
**Conversion of surgical methods**	0	0	
**Pain score** (Visual Analog Scale) Post-operative 12 h Post-operative 24 h	2.1 ± 2.4 1.9 ± 2.4	3.4 ± 2.7 2.8 ± 1.6	0.033 0.070
**Rescue analgesics requested on POD**^a^**1 ** None NSAIDs^a^ Meperidine	28 4 1	32 7 1	0.811
**Immediate post-operative complications**	0	0	
**Delayed post-operative complications**	1	0	

^a^ SPA-TLH, single-port access total laparoscopic hysterectomy; vNOTES-H, vaginal natural orifice transluminal endoscopic surgery; US, unilateral salpingectomy; BS, bilateral salpingectomy; USO, unilateral salpingo-oophorectomy; BSO, bilateral salpingo-oophorectomy; POD, postoperative day; NSAIDs, nonsteroidal anti-inflammatory drugs. ^b^ Hemoglobin on postoperative day 1 minus preoperative hemoglobin.

**Table 3:** Comparison of duration of each surgical procedure in minutes.

	vNOTES (*n* = 33)	SPA (*n* = 40)	*p*-Value
**Completion of port installation**	18.0 (14.0, 20.0)	5.0 (3.5, 5.5)	< 0.001
**Uterine artery ligation** ^a^ Right Left	N/A N/A	5.0 (3.0, 11.0) 5.5 (2.5, 10.5)	N/A N/A
**Adnexal surgery** Right Left	5.0 (2.5, 7.5) 3.0 (2.0, 5.0)	3.0 (1.5, 4.5) 4.5 (2.0, 6.0)	0.134 0.479
**Vesicovaginal space dissection**	N/A	5.8 ± 2.7	N/A
**Colpotomy**	N/A	5.0 (3.5, 9.0)	N/A
**Vaginal closure**	6.0 (4.0, 8.0)	12.5 (10.0, 16.0)	<0.001
**Bleeding control**	4.0 (2.5, 7.0)	4.0 (1.0, 9.5)	0.863
**Abdominal wound closure**	N/A	13.0 (10.0, 16.0)	N/A
**Total**	64.0 (46.0, 82.0)	82.0 (68.5, 121.0)	<0.001

^a^ In SPA surgery, uterine arterial ligation was done retroperitoneally, where the uterine artery diverges from the internal iliac artery.

**Table 4:** Patient and surgical information of the ureteral injury case.

	Information
**Age**	48
**Chief complaint**	Heavy menstrual bleeding, dysmenorrhea
**Parity**	Term delivery: 3 Preterm delivery: 0 Abortion: 1 Number of vaginal delivery: 3
**Menopausal status**	Pre-menopausal
**Medical/operative history**	None
**Pre-operative ultrasonography findings**	Size of the uterus: 9.23 cm × 6.48 cm (length x anterior-posterior) Number of leiomyoma: 1 Size of leiomyoma: 6.83 cm (longest diameter) Location of leiomyoma: posterior wall Fallopian tubes and ovaries: unremarkable
**Operative information**	Operation name: vNOTES^a^-hysterectomy and bilateral salpingectomy Operation time Completion of port installation: 20 min Cold knife morcellation: 15 min Bilateral salpingectomy: 8 min Bleeding control: 7 min Vaginal closure: 6 min Total: 76 min
**Intra-operative events**	None
**Events during hospitalization**	None
**Pre-operative hemoglobin levels (g/dl)**	13.9
**Post-operative hemoglobin levels (g/dl)**	10.0
**Post-operative urography CT ^a^ findings**	Profuse amount of fluid collection in the abdominal cavity. Contrast leakage at left distal ureter during the excretory phase. No hydronephrosis in both ureters. Mild peritoneal thickening, suggestive of peritonitis. Otherwise unremarkable.

^a^ vNOTES, vaginal natural orifice transluminal endoscopic surgery; CT, computed tomography.

## Data Availability

The data presented in this study are available on request from the corresponding author.
